# A Highlight on the Inhibition of Fungal Carbonic Anhydrases as Drug Targets for the Antifungal Armamentarium

**DOI:** 10.3390/ijms22094324

**Published:** 2021-04-21

**Authors:** Claudiu T. Supuran, Clemente Capasso

**Affiliations:** 1 Section of Pharmaceutical and Nutraceutical Sciences, Department of Neurofarba, University of Florence, Via U. Schiff 6, Sesto Fiorentino, 50019 Florence, Italy; 2 Institute of Biosciences and Bioresources, CNR, Via Pietro Castellino 111, 80131 Napoli, Italy

**Keywords:** carbon dioxide, carbonic anhydrases CO_2_-sensing, antifungals, CA inhibitors

## Abstract

Carbon dioxide (CO_2_), a vital molecule of the carbon cycle, is a critical component in living organisms’ metabolism, performing functions that lead to the building of compounds fundamental for the life cycle. In all living organisms, the CO_2_/bicarbonate (HCO_3_^−^) balancing is governed by a superfamily of enzymes, known as carbonic anhydrases (CAs, EC 4.2.1.1). CAs catalyze the pivotal physiological reaction, consisting of the reversible hydration of the CO_2_ to HCO_3_^−^ and protons. Opportunistic and pathogenic fungi can sense the environmental CO_2_ levels, which influence their virulence or environmental subsistence traits. The fungal CO_2_-sensing is directly stimulated by HCO_3_^−^ produced in a CA-dependent manner, which directly activates adenylyl cyclase (AC) involved in the fungal spore formation. The interference with CA activity may impair fungal growth and virulence, making this approach interesting for designing antifungal drugs with a novel mechanism of action: the inhibition of CAs linked to the CO_2_/HCO_3_^−^/pH chemosensing and signaling. This review reports that sulfonamides and their bioisosteres as well as inorganic anions can inhibit in vitro the β- and α-CAs from the fungi, suggesting how CAs may be considered as a novel “pathogen protein” target of many opportunistic, pathogenic fungi.

## 1. Introduction

The fossil fuel use, land-use changes as well as the natural carbon sources on land and in oceans has drastically influenced the growth rate of atmospheric CO_2_ [1]. In the last twenty years, human CO_2_ emissions have been enormously accelerated, considering the overall rise in energy consummation, the greater use of coal to produce energy, increased consumption per capita, and population development [1]. Changes in atmospheric CO_2_ mirrors the balance between carbon emissions due to human activity and the dynamics of many terrestrial and ocean processes that remove or emit CO_2_ [2]. The increased CO_2_ favors the photosynthetic activity of plants and increases carbon storage in the plants themselves and soil [2]. Cheng et al. believe that carbon storage seems to be mainly due to fungi (the so-called Arbuscular Mycorrhizal Fungi), which settle near the roots of about 80% of plant species, providing essential nutrients to the plants in exchange for carbohydrates [3]. CO_2_, a key molecule of the carbon cycle, is a critical component in the metabolism of living organisms, performing functions which lead to the building of compounds fundamental for the life cycle [4]. At the same time, CO_2_ is a waste product since it is the end-product of respiration, reaching a concentration of about 5% in the human bloodstream and tissues. This concentration is higher than the level of CO_2_ in the atmosphere (about 0.036%) [4]. Intriguingly, opportunistic and pathogenic fungi sense the CO_2_ difference, which influences fungal differentiation, determining the expression of those fungal features essential for virulent or non-virulent traits [5]. Pathogenic fungi are responsible for superficial diseases such as dermatophytes (infections of skin, hairs, and nails), or may lead to systemic illness (candidiasis, aspergillosis, cryptococcosis, mucormycosis, and others) [6,7,8,9]. Two molecules are crucial for the fungal CO_2_-sensing: (1) bicarbonate (HCO_3_^−^), which is a meiosis- and sporulation-promoting ion [10], and (2) adenylyl cyclase (AC) that is involved in the spore formation [11,12,13]. In *Cryptococcus*, bicarbonate directly activates a soluble form of AC necessary for the polysaccharide capsule formation [14,15,16,17]. AC catalyzes cyclic AMP (cAMP) synthesis, an essential intracellular regulatory molecule, which permits a link between CO_2_/HCO_3_^−^/pH chemosensing and signaling [18]. cAMP signaling is involved in many metabolic reactions as well as in fungal development and virulence [19]. The fungal virulence of *Cryptococcus neoformans*, the etiological agent responsible for cryptococcosis [9], is induced by high CO_2_ levels in mammalian hosts, causing the production of a massive polysaccharide capsule, which inhibits phagocytosis and impairs cell-mediated immune response [14,15,20]. However, it also true that other factors than high CO_2_ levels contribute to inhibit mating in the host as demonstrated by the use of a murine model of cryptococcosis. The carbonic anhydrase mutants for Can1 and Can2 (the two CAs encoded by the genome of *C. neoformans*) were both as virulent as wild type (wt), and quantitative measurements of fungal burden demonstrated that the Can2 mutant proliferates equivalently to the wt strain in the lungs and brain of infected animals [20]. In *Candida albicans*, the CO_2_ levels, through the relationship of bicarbonate, adenyl cyclase and cAMP, influence the growth of filamentous structures (hyphae), which are associated with the fungal virulence, adherence, secretion of hydrolases, and cell death in the hosts [19,21,22,23]. Twenty-six thiazolidines against several *Candida spp.* and Gram-positive and Gram-negative bacteria were tested. Although lacking significant antibacterial activity, the tested compounds exhibited selective antifungal activity with an equal potency to fluconazole and clotrimazole. Interestingly, CA was considered the putative target that could mediate the antifungal effects of these compounds [24].

### Fungal Enzymes Involved in the Bicarbonate Production (Physiological Role and Structural Features)

Cloning of the genomes of several pathogenic and non-pathogenic fungi provided the opportunity to identify a superfamily of ubiquitous metalloenzymes, known as carbonic anhydrases (CAs, EC 4.2.1.1), which catalyze a pivotal physiological reaction, consisting of the reversible hydration of the carbon dioxide to bicarbonate and protons [25,26,27,28,29,30,31]. The spontaneous reversible CO_2_ hydration reaction in the absence of the catalyst occurs very slowly with a rate constant of 0.15 s^−1^, which arrives at 50 s^−1^ for the reverse reaction of bicarbonate dehydration at the physiological pH [31]. CA increases the velocity of the CO_2_ hydration reaction up to 10^4^-10^6^-fold [31].

The CA superfamily is ubiquitously distributed in all living organisms and classified into eight CA classes (α, β, γ, δ, ζ, η, θ, and ι). Their distribution is quite varied from plants, animals, bacteria, and archaea. [25,26,27,28,29]. The genome of mammals, for example, encodes only for the α-CA class, of which 15 isoforms have been identified, which accomplish specialized functions in various tissues and organs [32,33,34,35,36]. In plants, α and β-CAs actively participate in photosynthesis and biosynthetic reactions associated with it, as well as in some aforementioned processes [37]. In Bacteria, Archaea, and cyanobacteria, α, β, γ, and ι -CA classes are present. Their role is to balance the CO_2_/HCO_3_^−^ concentration ratio and a role in the carbon dioxide fixation [29,30,31,37,38,39]. Marine diatoms encode for α- δ-,ζ-, θ- and ι-CAs, which are involved in carbon dioxide fixation and metabolism [40,41,42]. In protozoa have been detected α- and η-CAs. Probably, the η-CA-class, recently discovered, has a pivotal role in de novo purine/pyrimidine biosynthetic pathways [43]. 

The fungal CO_2_-sensing, related to the CO_2_/HCO_3_^−^/pH-sensing, is directly stimulated by HCO_3_^−^ produced in a CA-dependent manner. In the fungal kingdom, the typical CA class identified is represented by β-class, and the majority of fungi encode at least one β-CA [13,44,45]. The genomes of basidiomycetous and hemiascomycetous yeasts encode only for β-CAs. In contrast, most filamentous ascomycetes contain multiple β-CA genes and, in some of them, it is possible to find genes encoding for α-CAs [13,44,45]. Here, some examples demonstrating that CAs are abundant in fungi and yeasts (the last are microscopic fungi consisting of solitary cells that reproduce by budding) as reported in the following examples. *Saccharomyces cerevisiae*, *Candida albicans*, and *Candida glabrata* have only one β-CA, whereas multiple copies of β-CA and α-CA-encoding genes were reported in other fungi [44,45]. Recently, it has been evidenced that CAs play an important role in fungal pathogen sensing and the control of sexual growth [44,45]. The β-CAs identified in *Candida albicans* and *Candida glabrata* indicated with the acronyms CaNce103 and CgNce103, respectively, are necessary for the development of these fungi in environments characterized by low-oxygen conditions, such as the skin [44,45]. The CA (Can2) encoded by the genome of *Cryptococcus neoformans* allows the growth of the yeast in its natural habitat. It is relevant to note how the link between AC, cAMP signaling, and CO_2_/HCO_3_^−^ sensing is conserved in most fungi since it is an essential mediator of fungal metabolism and pathogenesis [13,44,45]. Again, the gene Nce103 identified in the genome of *Saccharomyces cerevisiae* encodes for a β-CA (ScCA), which is involved in the production of the bicarbonate essential for the enzyme catalyzing carboxylation reactions, such as the pyruvate carboxylase (PC), acetyl-CoA carboxylase (ACC), carbamoyl phosphate synthase (CPSase), and phosphoribosylaminoimidazole (AIR) carboxylase [46,47]. 

In 2009, the first crystal structure of the β-CA encoded in the genome of a fungus, i.e., *Cryptococcus neoformans*, was reported by Schlicker and coworkers [45]. It showed a dimeric organization similar to that found in the CA belonging to the plant-type β-class (the two cysteines and a histidine responsible for zinc coordination are conserved in the active site of such enzymes). Intriguingly, a Can2 (acronym used for the CA from *C. neoformans*) three-dimensional structure showed a peculiar N-terminal extension, which interacts with the entrance of the catalytic pocket of the dimer. The N-terminus is an internal regulator or an interaction site for a regulatory protein, affecting the Can2 activity [45]. It can be considered a switch for the activation/inactivation of the protein, which is regulated by physiological factors, like pH, small molecule, or proteins. [45]. 

In 2011, the structure of the first fungal α-CA was obtained, which was identified in the fungus *Aspergillus oryzae* [48]. Like for other α-CAs, the enzyme showed a central core formed by a twisted β sheet consisting of eight mostly anti-parallel strands. The ion cofactor resulted in an atom of Zn(II) coordinated to the three histidines of the catalytic pocket, which is at the bottom of a deep cavity in the protein center [48]. 

In 2014, the structures of two β-CAs belonging to the fungus *Sordaria macrospora* were resolved by X-ray crystallography [49]. Like Can2, the two β-CAs from *S. macrospora* showed a high structural similarity with plant-like β-CAs, but it was assembled in a tetrameric and not a dimeric form. The two CAs (CAS1 and CAS2) were distinguished for the type of conformations they assumed: CAS1 resulted in the open “type- I” conformation, while the CAS2 adopted a close “type-II” conformation [49]. Finally, between 2020 and 2021, CafA and CafB, two of the four β-CAs encoded by the genome of the fungus *Aspergillus fumigatus*, were crystallized and the structure resolved at 1.8 and 2.0 Å, respectively [50,51]. The catalytic sites of CafA and CafB look similar to those of other β-CAs. CafA showed the typical open conformation. Surprisingly, CafB revealed a unique active site at a low pH or in an oxidative environment, resulting in an inactive enzyme, with a disulfide bond formed by the two zinc-ligating cysteines [50]. Of course, CafB also adopts the typical active/inactive configurations in which a conserved aspartic acid is implicated in switching the enzyme in its open/closed state [52].

This review reports the recent kinetic investigations and inhibition profiles obtained for the fungal CAs encoded in the genomes of *Sordaria macrospora, Saccharomyces cerevisiae*, *Candida glabrata*, *Malassezia restricta,* and *Malassezia globosa*. As described above, it is readily apparent how fungal CAs could play an essential role in the life cycle of opportunistic and pathogenic fungi. It is reasonable to think that the interference with their activity may impair the fungal growth and virulence, making this approach interesting for designing antifungal drugs with a novel mechanism of action that consists in the inhibition of the CA system linked to CO_2_/HCO_3_^−^/pH chemosensing and signaling. The inhibition of the CAs from pathogens represents an essential aspect in fighting the drug-resistance problems developed by many pathogenic microorganisms, whose growth could be impaired through the CA inhibition. We suggested that the activity of CAs is connected to the microbial survival because their activity supports many physiological microorganism functions, which require inorganic carbon [31]. In fungi, the CA inhibition affects the CO_2_/HCO_3_^−^ balancing pathway impairing their growth as it happens in bacteria. It has also been demonstrated that carbonic anhydrase inhibitors (CAIs) could inhibit the growth of *M. globosa*, *C. albicans*, *C. neoformans* in vivo in conditions of limited CO_2_ availability (i.e., the skin surface infected by the fungus) [53]. Species of the genus Malassezia are the most abundant fungi of the skin characterized by a CO_2_ level very similar to ambient air. A causative link between Malassezia and disease pathogenesis remains unknown since there is a lack of information on the complex interaction of Malassezia with the immune system of the skin [54]. For this reason, an experimental model of Malassezia skin infection in mice was recently established to investigate the interaction of the fungus with the skin immune system in the context of homeostasis and disease [54]. 

The inhibitors towards the CAs identified in pathogens could improve the chemical arsenal used to contrast the drug-resistance phenomenon. Fortunately, many CAIs exist, as reported in the next paragraph. Therefore, these enzymes could be validated as a “pathogen protein” target, as demonstrated by the recent developments achieved in the field. Recently, FDA-approved carbonic anhydrase inhibitors, such as acetazolamide, methazolamide, and ethoxzolamide, were opportunely modified to target the bacterial CAs of the vancomycin-resistant enterococci (VRE) [55], and a similar approach may be used to develop antifungal agents. In fact, it has been reported that 3 mM ethoxyzolamide (a classical CA inhibitor) significantly reduced the growth of *C. neoformans* in 0.033% CO_2_, while, as expected, 5% CO_2_ reestablishes its growth [44]. These results confirm that ethoxyzolamide inhibits intracellular carbonic anhydrase activity in *C. neoformans,* and that the pathogen requires CA activity for growing in ambient air concentrations of CO_2_. Interestingly, the addition of fatty acids can restore the growth of Can2 mutants. This suggests that the lack of bicarbonate production by Can2 mutants affects fatty acid synthesis, causing a growth defect. Interestingly, *C. neoformans* virulence, which develops during systemic infection characterized by high CO_2_ concentration, is dependent on the Can2 activity, suggesting the existence of a bicarbonate-dependent signaling cascade [44]. It has been proposed that CO_2_ diffuses into the cell. Here, it is hydrated to bicarbonate by Can2 when present in limiting concentrations. HCO_3_^−^ stimulates adenylyl cyclase activity, resulting in the activation of the cAMP-signaling pathway, which controls significant virulence determinants such as capsule biosynthesis [44].

## 2. Main Class of CA Inhibitors (Sulfonamides and Anions)

### 2.1. Substituted Benzene-Sulfonamides 

**Figure 1:**
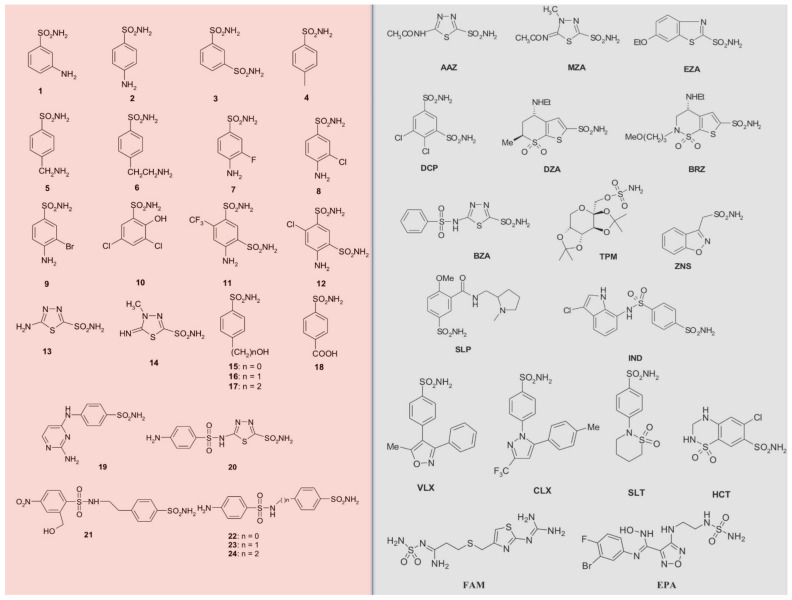
Sulfonamide/sulfamate/sulfamide of types **1**–**24** (pink background) and **AAZ-EPA** (gray background) investigated as fungal CA inhibitors. Legend: **AAZ**, acetazolamide; **MZA,** methazolamide; **EZA,** ethoxzolamide; **DCP**, dichlorophenamide; **DZA,** dorzolamide; **BRZ**, brinznolamide; **BZA,** benzolamide; **TPM,** topiramate; **ZNS**, zonisamide; **SLP**, sulpiride; **IND,** indisulam; **VLX,** valdecoxib; **CLX,** celecoxib; **SLT,** sulthiame; **HCT,** hydrochlorothiazide; **FAM,** famotidine; **EPA**, epacadostat.

The first antimicrobial drug widely used in clinical settings was Prontosil [56], a sulfanilamide prodrug, which is isosteric/isostructural with p-aminobenzoic acid (PABA), the substrate of dihydropteroate synthase (DHPS) [57,58]. After sulfanilamide, a range of analogs, the sulfa drug class, are still used as antibacterials, even if many of them show substantial drug resistance issues. Sulfa drugs are derived from sulfonamides, and the presence of primary sulfonamide moieties in sulfanilamide characterized most of the investigated CAIs until recently [32,59,60,61]. Primary sulfonamides/sulfamates/sulfamides possess the general formula R-X-SO_2_NH_2_, where R can be an aromatic, heterocyclic, aliphatic, or sugar scaffold, X = nothing, O or NH (Figure 1). 

Most of the sulfonamides acting as CAIs bind Zn (II) in a tetrahedral geometry, showing an extended network of hydrogen bonds with the enzyme amino acid residues, as seen by the enzyme-inhibitor X-ray crystallographic data [32,61,62]. The aromatic/heterocyclic part of the inhibitor interacts with the hydrophilic and hydrophobic residues of the catalytic cavity [32,61]. Compounds containing -SO_2_NH_2_ group, including clinically licensed drugs, are generally considered CAIs [27,63,64,65,66,67,68,69,70,71,72,73,74,75,76,77,78]. Some examples include: **AAZ**, **MZA**, **EZA**, and **DCP**, which are systemically acting antiglaucoma CAIs; **DZA** and **BRZ** are antiglaucoma agents; **BZA** belongs to the same pharmacological class; **ZNS**, **SLT**, and **TPM** are antiepileptic drugs; and **SLP** and **IND**, with COX2 selective inhibitors **CLX** and **VLX**. The diuretic hydrochlorothiazide (**HCT)** is also known to act as a CAI [35,79,80]. **FAM** is a competitive histamine H2-receptor antagonist [79], and **EPA** is an inhibitor of the heme-containing enzyme, indoleamine 2,3-dioxygenase-1 (IDO1), but they also act as CAIs [80] (see Figure 1). Table 1 shows selected inhibition data with some of these compounds against selected fungal CAs.

### 2.2. Inorganic Metal-Complexing Anions or More Complicated Species 

These CA inhibitors include inorganic anions as well as several more complex species such as carboxylates, which are in fact organic anions [61,62]. Anions may bind either in the tetrahedral geometry of the metal ion or as trigonal–bipyramidal adducts [86]. Anion inhibitors show K_Is_ in a millimolar range, diversely from the sulfonamides mentioned above, which is generally showed as K_Is_ in the micro to nanomolar range. But their investigation as CA inhibitors offers the possibility to better understand the inhibition/catalytic mechanisms of the CAs, for improving the design of novel types of inhibitors that may have clinical applications [61,62]. A list of anions and their CA inhibitory action against selected fungal CAs is shown in Table 2.

**Figure 2:**
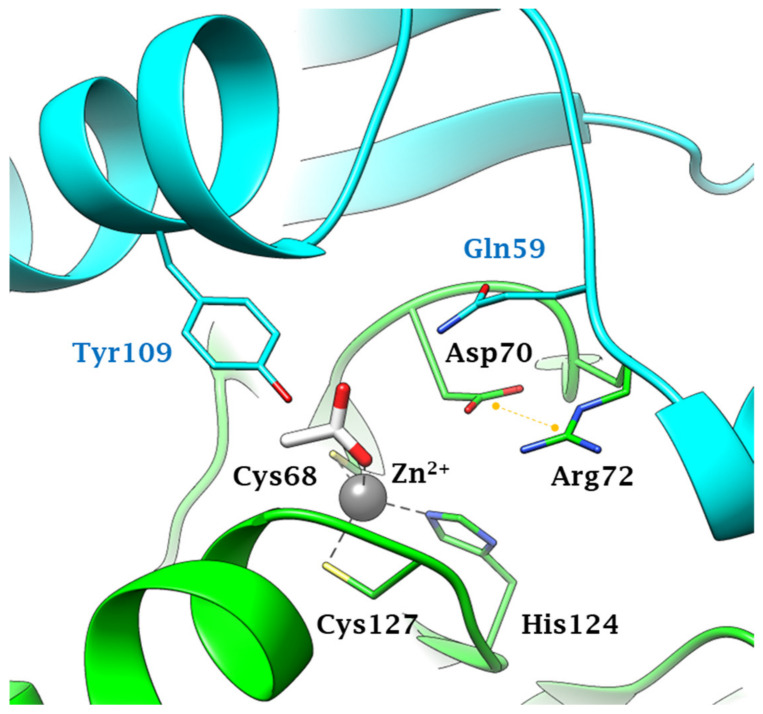
Active site view of Can2 (pdb 2W3N) complexed to the anion inhibitor acetate [45]. Protomers A and B are colored green and cyan respectively. Residues from protomers A and B are labeled black and light blu, respectively. The Zn^2+^ ion, represented as a grey sphere, is coordinated by two cysteines and one histidine residue from monomer A and by one acetate ion as a ligand. The salt bridge in the Asp-Arg dyad is represented as a yellow dashed line.

Anions inhibit fungal CAs by coordinating to the metal ion within the enzyme active site, as exemplified in Figure 2 for Can2 complexed with acetate. As for all β-CAs, Can2 is a dimer and the active site contains amino acid residues from both monomers. The zinc ion is coordinated as shown in Figure 2, by Cys68, His124, and Cys127, whereas acetate is the fourth zin ligand, being coordinated monodentately by one of the oxygen atoms. The same type of inhibition mechanism is valid for all anions shown in Table 2, although few X-ray crystal structures of such adducts are available to date [45].

## 3. Other Classes of Less Investigated CAIs 

### 3.1. Dithiocarbamates

Dithiocarbamates (DTCs) represent another class of CAIs [89,90,91,92,93,94]. They were discovered by considering the inorganic anion trithiocarbonate (TTC, CS_3_^2−^) as a lead compound [95]. DTCs, as TTC, coordinate through one sulfur atom to the Zn(II) ion from the enzyme active site and also interact with the conserved Thr199 amino acid residue. Besides, DTC organic scaffolds participate in supplementary interactions with the enzyme active site [33,91,96,97]. However, there are no X-ray crystal structures of fungal CAs with DTC inhibitors reported to date.

### 3.2. Phenols

Other than sulfonamides and their isosteres, as well as anions and DTCs, CAs are also inhibited by phenols, which anchor to the water molecule/hydroxide ion coordinated to the metal ion [62,86,98]. Again, no structural work on phenolic fungal CAIs were reported for the moment, and these compounds are not discussed in detail here.

## 4. Kinetic Parameters and Inhibition Profiles of Various Fungal CAs

### 4.1. Saccharomyces Cerevisiae CA

*Saccharomyces cerevisiae* has been used as a model organism since its high degree of similarity of the biological processes with the human cells [99]. Since it can be easily manipulated, *S. cerevisiae* was used to develop novel antifungals, such as those altering the mitochondrial functions or inducing oxidative damage [100]. In the genome of *S. cerevisiae*, the gene Nce103, encoding for a β-CA, designated as scCA, has been identified. It provides the bicarbonate essential to the metabolic carboxylation reactions of the yeast metabolism [101]. scCA is an efficient catalyst for CO_2_ hydration to bicarbonate and protons, with kinetic parameters as follows, k_cat_ of 9.4 × 10^5^ s^−1^ and k_cat_/K_M_ of 9.8 × 10^7^ M^−1^s^−1^.

#### 4.1.1. Sulfonamide Inhibition

scCA inhibition with sulfonamides has been investigated. The clinically used sulfonamides/sulfamates, such as acetazolamide, ethoxzolamide, methazolamide, dorzolamide, topiramate, celecoxib, and others, generally showed effective scCA inhibitory activity, with K_Is_ in the range of 82.6–133 nM [102]. Moreover, Benzenesulfonamides substituted in 2-, 4- and 3,4-positions with amino, alkyl, halogen, and hydroxyalkyl moieties had K_Is_ in the range of 0.976–18.45 μM [102]. Lower K_Is_ (154—654 nM) were observed for benzenesulfonamides incorporating aminoalkyl/carboxyalkyl moieties or halogenosulfanilamides; benzene- 1,3-disulfonamides; simple heterocyclic sulfonamides and sulfanilyl-sulfonamides. K_I_ of 15.1 nM was obtained for 4-(2-amino-pyrimidin-4-yl)-benzenesulfonamide [102]. 

#### 4.1.2. Anion Inhibition

This class of inorganic metal-complexing anions can bind to the metal ion within their enzyme active site, interfering with the enzymatic catalytic process. The anion inhibitors with lower K_Is_ (8.7–10.8 μM) were bromide, iodide, and sulfamide [102].

#### 4.1.3. Dithiocarbamate Inhibition 

DTCs are a relatively new class of CAIs. They are usually obtained by the reaction of primary or secondary amines with carbon disulfide, and most of these derivatives incorporated alkyl, mono-/bicyclic aliphatic, and heterocyclic rings but also heterocycles such as piperidine, morpholine, and piperazine. They were investigated for their inhibitory activity against scCA [103]. Some of these DTCs resulted in low nanomolar activity against the yeast enzyme (K_IS_ = 6.4 and 259 nM) [103]. Intriguingly, several of the investigated DTCs showed excellent selectivity ratios for inhibiting scCA over the human cytosolic CA isoforms (hCA I inhibitors with K_Is_ of 66.5–910 nM, hCA II inhibitors with K_Is_ of 8.9–107 nM) [103].

#### 4.1.4. Phenols as Inhibitors

A series of phenols incorporating tertiary amine and trans-pyridylethenyl-carbonyl moieties were assayed as inhibitors of scCA [104]. One of these compounds was a low nanomolar scCA inhibitor, whereas the remainder inhibited the enzyme with K_Is_ in the range of 23.5–95.4 nM [104]. The human isoforms hCA I and hCA II were less sensitive to inhibiton by phenols, since the K_Is_ were of 0.78–23.5 µM against hCA I and of 10.8–52.4 µM against hCA II [104]. 

### 4.2. Candida Glabrata CA

*Candida glabrata* is a haploid yeast belonging to the genus Candida and is considered the most common cause of candidiasis [105]. One of the significant obstacles in infections caused by *C. glabrata* is its innate resistance to azole antimycotic therapy, which is very effective in treating infections caused by other Candida species [105]. This pathogenic fungus encodes for a β- CA, indicated with the acronym CgNce103 or CgCA. The enzyme showed significant CO_2_ hydrase activity, with a k_cat_ of 3.8 × 10^5^ s^−1^ and k_cat_/K_M_ of 4.8 × 10^7^ M^−1^ s^−1^ [106].

#### 4.2.1. Sulfonamide Inhibition

Most simple sulfonamides showed weak or moderate CgNce103 inhibitory properties. In contrast, acetazolamide and a series of 4-substituted ureido-benzene-sulfonamides, sulfamates and sulfamides effectively inhibited CgNce103 with K_Is_ in the range of 4.1–115 nM, whereas many such compounds were ineffective, such as hCA II inhibitors. As *C. glabrata* offers significant resistance to many classical antifungal agents, sulfonamide inhibition of CgNce103 may allow an exciting means for limiting pathogen growth, leading to the development of antifungals with a novel mechanism of action. Besides, four generations (G0-G3) of poly(amidoamine) (PAMAM) dendrimers incorporating benzenesulfonamide moieties were investigated as inhibitors of CgNce103 [81]. The enzyme was efficiently inhibited by the four generations PAMAM–sulfonamide dendrimer with K_IS_ = 66–509 nM [81]. CaNce103 from *C. albicans* and CgNce103 from *C. glabrata* were investigated for their inhibition with structurally novel isatin-containing sulfonamides, too [82]. The compounds show K_I_ values in the low nanomolar range and significantly higher K_I_ values for the human CAs. Unfortunately, no crystal structures are available for both enzymes, and homology models were constructed to rationalize their enzyme inhibition values. Using the obtained homology models, it has been seen that the backbone fold of the two enzymes showed marked differences near the active site, especially in the region where the nitro group is most likely located [82]. CaNce103 was investigated for its inhibition with A series of novel sulfamides incorporating the dopamine scaffold. CaNce103 was inhibited in the low micromolar to nanomolar range by the dopamine sulfamide analogues [107]. Finally, the CA from *C. glabrata* was inhibited by a series of 6-substituted benzoxaboroles in the nanomolar range, demonstrating that benzoxaborole chemotype may offer exciting development opportunities of antifungal agents [87]. 

#### 4.2.2. Anion Inhibition

Investigation into its inhibition with a series of simple inorganic anions such as halogenides, pseudohalogenides, bicarbonate, carbonate, nitrate, nitrite, hydrogen sulfide, bisulfite, perchlorate, sulfate, and some isosteric species showed that CgNce103 was moderately inhibited by metal poisons, such as cyanide, azide, cyanate, thiocyanate (K_Is_ of 0.60–1.12 mM), and was strongly inhibited by bicarbonate, nitrate, nitrite, and phenylarsonic acid (K_Is_ of 86–98 μM) [106]. The other anions showed inhibition constants in the low millimolar range, except for bromide and iodide (K_Is_ of 27–42 mM) [106].

### 4.3. Sordaria Macrospora CAs

Four different CA-genes indicated with the acronyms *cas1*, *cas2*, *cas3*, and *cas4* are encoded in the genome of the filamentous ascomycete *Sordaria macrospora,* which is considered to be a model organism in biology, like *S. cerevisiae* [83]. The proteins encoded by these genes are indicated as CAS1, CAS2, CAS3, and CAS4 [49,84,108]. CAS1 and CAS2 are strictly related proteins that belong to the plant-like subgroup of β-CAs, CAS3 encodes a cab-type β-CA (cab is the β-CA purified from *Methanobacterium thermoautotrophicum* [109]), while CAS4 is an α-CA. CAS1 and CAS3 are localized in the cytoplasm, whereas the amino acid sequence of CAS2 is characterized by a signal peptide responsible for its translocation into the mitochondria, diversely from the α-CA CAS4, which was assumed to be a secreted protein [49,84,108]. It has been demonstrated that CAS1 and CAS2 are involved in the bicarbonate-dependent regulation of fruiting body development. CAS2 regulates hyphal growth and germination, too. Intriguingly, the deletion of one of the two—CAS1 or CAS2—is not lethal for the microorganism [13]. CAS1 and CAS2 showed a low activity for the CO_2_ hydration reaction with a k_cat_ = 1.2 × 10^4^ s^−1^ and a k_cat_ = 1.3 × 10^4^ s^−1^, respectively [49]. CAS3 had a higher activity level, showing an order of magnitude higher than catalytic activity (k_cat_ = 7.9 × 10^5^ s^−1^) for the CAS1 and CAS2. CAS4 has not yet been investigated up to now. Interestingly, CAS3 activity resulted in the same order as those calculated for the β-CAs from *Cryptococcus neoformans*, *Candida albicans,* and *Saccharomyces cerevisiae* [110]. 

#### 4.3.1. Sulfonamide Inhibition 

A panel of aromatic compounds, including heterocyclic, aliphatic sulfonamides, and one sulfamate, were tested to determine their inhibition constants for the related enzymes, CAS1, CAS2, and CAS3 [111,112]. CAS1 was efficiently inhibited by simple aromatic sulfonamides, such as tosylamide, 3-fluoro-/chloro-sulfanilamide, which has inhibition constants in the range of 43.2–79.6 nM. Heterocyclic derivatives such as acetazolamide, methazolamide, topiramate, ethoxzolamide, dorzolamide, and brinzolamide showed medium potency inhibitory action with inhibition constants of 360–445 nM [112]. CAS2 was, on the other hand, less sensitive to inhibition with sulfonamides. However, some effective CAS2 inhibitors comprised 5-amino-1,3,4-thiadiazole-2-sulfonamide, which is in fact the deacetylated precursor of the classical sulfonamide acetazolamide. 4-Hydroxymethyl-benzenesulfonamide was also an effective inhibitor. These two compounds showed inhibition constants of 48.1–92.5nM against CAS2 [112]. Acetazolamide, dorzolamide, ethoxzolamide, topiramate, sulpiride, indisulam, celecoxib, and sulthiame were medium potency CAS2 inhibitors (K_Is_ of 143–857 nM) [112]. The most effective CAS3 inhibitors were benzolamide, brinzolamide, dichlorophenamide, methazolamide, acetazolamide, ethoxzolamide, sulfanilamide, methanilamide, and benzene-1,3-disulfonamide, with K_Is_ in the range of 54–95 nM [111]. CAS3 generally showed a higher affinity for sulfonamide inhibitors compared to CAS1 and CAS2 [111]. Other sulfonamides showed affinities in the high micromolar range or were ineffective as CAS1/2/3 inhibitors [111,112]. Intriguing, small changes in the inhibitor structure led to important differences in the three enzymes’ activity, demonstrating that it is possible to tune the enzyme inhibition, altering the inhibitor scaffold of the inhibitors [49,111,112]. This makes possible the synthesis of selective inhibitors, which may interfere with the activity of the fungal CAs, leaving unaltered the activity of the human α-CAs.

#### 4.3.2. Anion Inhibition

Up to now, only CAS3 has been investigated for its inhibition profiles with this class of inorganic metal-complexing anions, which can bind to the metal ion within their enzyme active site, interfering with the enzymatic catalytic process. The most effective CAS3 anions/small molecule inhibitors were diethyl-dithiocarbamate, sulfamide, sulfamate, phenyl boronic, and phenyl arsonic acids, with K_Is_ in the range of 89–97 μM [110]. Anions such as iodide, the pseudohalides, bicarbonate, carbonate, nitrate, nitrite, hydrogensulfide, stannate, selenate, tellurate, tetraborate, perrhenate, perruthenate, selenocyanide, and trithiocarbonate resulted in low millimolar CAS3 inhibitors [110]. CAS3 was not inhibited by halides, sulfate, hydrogensulfite, peroxydisulfate, diphosphate, divanadate, perchlorate, tetrafluoroborate, fluorosulfonate, and iminodisulfonate [110].

### 4.4. Malassezia Globosa and M. Restricta 

Malassezia is a fungi genus, including seven species: *M. furfur*, *M. pachydermatis*, and *M. sympodialis*, *M. globosa*, *M. obtusa*, *M. restricta*, and *M. slooffiae*. 

*M. globosa* have been identified as members of the human cutaneous flora, coexisting with the skin’s microbial flora [113]. Malassezia fungi represent the etiologic agents of specific skin diseases, such as pityriasis versicolor, seborrheic dermatitis scalp, and dandruff [113]. The last condition is a frequent skin disorder restricted to the scalp. It is caused by the oleic acid of the scalp sebum, produced through t hydrolysis of triglycerides, which are accomplished by the lipases produced mainly by *M. globosa* [114]. Other than *M. globose*, another fungus, *M. restricta,* is also involved in starting the disequilibrium between the commensals *Cutibacterium acnes* (formerly named *Propionibacterium acnes*) and *Staphylococcus* sp., both of which contribute to dandruff and seborrheic dermatitis symptoms [85,115,116]. Most strategies used to treat the dandruff are based on impairing the growth of the fungi mentioned above, using active ingredients in anti-dandruff shampoos, such as the ketoconazole B, an azole antifungal agent interfering with the biosynthesis of fungal sterols, or the pyridinethione A that interferes with the synthesis of ergosterol, a key component of fungal cell walls [117,118]. However, the efficacy of these compounds in preventing/treating dandruff is not very high. Thus, it is necessary to target new molecules of the Malassezia metabolism, such as the carbonic anhydrases, recently studied as protein-fungal targets by our groups.

#### 4.4.1. Malassezia globosa CA

The genome of the fungal parasite *Malassezia globosa* contains a single gene encoding a CA (acronym MgCA) belonging to the β-class. The enzyme showed an appreciable CO_2_ hydrase activity, with a k_cat_ of 9.2 × 10^5^ s^−1^ and k_cat_/K_M_ of 8.3 × 10^7^ M^−1^ s^−1^ [119].

##### Sulfonamide Inhibition 

Many primary sulfonamides showed K_Is_ in the nanomolar range of 63–174 nM. The clinically used drugs belonging to the series **AZZ-HTC**, such as **MZA**, **EZA**, **BRZ**, **CLX**, and **SAC**, acted as mild inhibitors of MgCA (K_Is_ = 31.5–79 μM) [120]. Interesting to note, in general, MgCA potent inhibitors resulted in mild inhibitors of the β-CA from *M. restricta* and vice-versa, highlighting how different the inhibition pattern of the two homologous fungal enzymes was [120]. Again, the two fungal enzymes had an inhibition profile highly distinct from those of the two human isoenzymes [120]. 

##### Anion Inhibition 

Anions, such as halides, pseudohalides, nitrite/nitrate, sulfite/sulfate and anions isoelectronic with them, but also complex anions incorporating heavy metals, as well as the simple small molecules known to have an affinity for Zn (II) in the CAs, such as sulfamide, sulfamic acid, phenylboronic and phenylarsonic acid, bicarbonate and diethyldithiocarbamate, have been known for their interaction with MgCA. The best MgCA inhibitors were sulfamide, sulfamate, phenylboronic acid, phenylarsonic acid, bicarbonate, and diethyldithiocarbamate, with K_Is_ ranging between 83 and 94 µM [119]. Interestingly, bicarbonate is also a substrate/reaction product of the CAs, and this behavior is entirely unexpected, especially considering that carbonate did not show inhibitory properties [119]. This inhibitory behavior of the enzyme, which is very difficult to explain, could be physiologically crucial.

##### Dithiocarbamate Inhibition 

A series of DTCs, incorporating various scaffolds, among which are those of N,N-dimethylaminoethylenediamine, the amino alcohols with 3–5 carbon atoms in their molecule, 3-amino-quinuclidine, piperidine, morpholine and piperazine derivatives, as well as phenethylamine and its 4-sulfamoylated derivative, were investigated for the inhibition of MgCA [121]. Most of the DTCs were shown to be effective, although not low, nanomolar potencies against MgCA, but several of them showed a with K_Is_ ranging between 383 and 6235 nM, resulting in very effective inhibitors of MgCA when compared with the standard sulfonamide drug acetazolamide (K_I_ of 74 µM) [121]. As a result, DTCs could be critical molecules in searching for more potent and efficient fungal CAIs.

##### Monothiocarbamate Inhibition 

Monothiocarbamates (MTCs) were used to inhibit MgCA. They incorporate different scaffolds, among which were aliphatic amine with 1–4 carbons atom in their molecule, morpholine, piperazine, as well as phenethylamine and benzylamine derivatives. The considered MTCs showed K_Is_ spanning between 1.85 and 18.9 µM, producing results better than those observed for the clinically used sulfonamide drug acetazolamide (K_I_ of 74 µM) [88]. The docking studies applied to the homology model of MgCA have highlighted the main differences in the binding mode of MTCs and DTCs within the fungal CA active site [88]. 

##### Phenols as Inhibitors

A panel of 22 phenols was investigated as inhibitors of MgCA. All tested phenols possessed a better efficacy in inhibiting MgCA than the clinically used sulfonamide acetazolamide, with K_Is_ in the range of 2.5 and 65.0 µM [122]. A homology-built model of MgCA revealed a vast network of hydrogen bonds and hydrophobic interactions between the phenol and active site residues. The OH moiety of the inhibitor was anchored to the zinc-coordinated water, making hydrogen bonds with Ser48 and Asp49 in the catalytic pocket [122]. MgCA was also investigated for its inhibition profile with various natural phenols, such as xanthomicrol and rosmarinic acid, which inhibited MgCA with a K_IS_ = 0.6 and 2.2 µM, respectively [123]. 

##### Phosphonamidates

A phosphorus-based zinc-binding motif, such as phosphonamidate, inhibited the MgCA with K_Is_ in the range 28–256 µM (Can2, CA from *C. neoformans,* with K_Is_ = 50–3488 µM and CgNce, CA from *Candida glabrata,* with K_Is_ = 23–656 µM) [124]. Interestingly, the human isoforms (CAs I and II) are inhibited in a high micromolar range (32.8–961.2 mM), suggesting that this group of inhibitors could be considered attractive leads for developing new anti-infective agents. 

#### 4.4.2. Malassezia restricta CA

The genome of the opportunistic pathogen *Malassezia restricta* encodes for a β-CA (acronym MreCA), which had a high catalytic activity for the hydration of CO_2_ into bicarbonate and protons, with the following kinetic parameters: k_cat_ = 1.06 × 10^6^ s^−1^ and k_cat_/K_M_ = 1.07 × 10^8^ M^−1^s^−1^ [125].

##### Sulfonamide Inhibition 

The investigation of the sulfonamide inhibition profile of MreCA provides evidence that the MreCA sulfonamide inhibitors, such as dorzolamide, brinzolamide, indisulam, valdecoxib, sulthiam, and acetazolamide, inhibited the enzyme with a K_I_ < 1.0 μM [120]. These inhibitors resulted in the best for MreCA. Noticeably, the MreCA sulfonamide inhibition profile was very different from those of the homologous enzyme MgCA and the human isoenzymes, hCA I and hCA II [120]. The crystallographic structures of MreCA and MgCA not being available, it has been speculated that the fungal and the human CAs (hCA I and hCA II) have a similar catalytic site, but unusual architectural features, which may be responsible for the differences in K_Is_ obtained for the two fungal and human enzymes. 

##### Anion Inhibition 

The investigation of the classical anions with their inhibition for MreCA showed that the most effective were diethyldithiocarbamate, sulfamide, phenyl arsenic acid, stannate, tellurate, tetraborate, selenocyanate, trithiocarbonate, and bicarbonate [126]. Moreover, in this case, MreCA and MgCA resulted in different K_Is_ for the inhibitors used [126]. It is possible that the differences in the catalytic site could be the cause of their peculiar inhibitory behavior. All this justifies why the anion inhibition profile studies are crucial for the comprehension of the CA inhibition/catalytic mechanisms, allowing the design of novel types of inhibitors, which may have clinical applications for the management of dandruff and seborrheic dermatitis.

## 5. Conclusions

Drug-resistant microorganisms are an unavoidable and everyday phenomenon that requires the search for new anti-infectives with a novel mechanism of action. CAs have only recently begun to be studied in depth in microorganisms, since it has been shown that CAs are essential for the life cycle of many pathogens, and their inhibition may lead to their growth deficiency. Many opportunistic or pathogenic fungi, such as *C. albicans*, *C. glabrata*, *C. neoformans*, *M. globosa*, *M. restricta*, *S. cereviasiae*, and many others, encode for β-CAs, which, with their activity, are involved in the production of bicarbonate, an adenyl cyclase inducer. The CA/adenyl cyclase system constitutes the mechanism of CO_2_-sensing which can be deregulated by inhibiting one of these enzymes. Interesting, some fungi also present α-CAs, which are less investigated concerning the β-class since the α-CAs are rarer to find. The current review reports that sulfonamides and their bioisosteres can inhibit in vitro the β- and α-CAs from the fungi aforementioned above. Moreover, it has also been demonstrated that these compounds could inhibit the growth of *M. globosa*, *C. albicans*, and *C. neoformans* in vivo in conditions of limited CO_2_ availability (i.e., the skin surface infected by the fungus) [53]. This evidence corroborates how the fungal CAs could be considered druggable targets, mainly because there are no β-class enzymes in humans, and thus, the side effects due to inhibition of the host enzymes are not significant. In conclusion, the tendency to selectively inhibit CAs from pathogens such as fungi might constitute an alternative approach for designing anti-infectives with novel mechanisms of action. This is a rather new research field, which requires many other X-ray crystal structures of CAs from pathogens (as very few of them are presently available) together with massive drug design campaigns for finding selective inhibitors of the pathogenic versus the host enzymes.

## Figures and Tables

**Table 1:** Inhibition data of human isoenzymes (CA I and CA II) and fungal CAs (MreCA, MgCA, CAS1, CAS2, CAS3; Figure 1) by a stopped-flow CO_2_ hydrase assay. The other fungal CAs have been previously reviewed by Elleuche and Poggeler (see references [81,82]).

Inhibitor	K_I_ (nM) *						
	hCA I ^1^ (α-CA)	hCA II ^1^ (α-CA)	MgCA ^2^ (β-CA)	MreCA ^2^ (β-CA)	CAS1 ^1^ (β-CA)	CAS2 ^1^ (β-CA)	CAS3 ^1^ (β-CA)
**1**	28,000	300	980	412	361	386	90
**2**	25,000	240	24.5	462	144	3480	84
**3**	79	8	15.2	>10,000	225	3630	83
**4**	78,500	320	674	404	47.1	6900	560
**5**	25,000	170	17.4	>10,000	323	8720	726
**6**	21,000	160	7.9	>10,000	241	7650	441
**7**	8300	60	11.6	459	43.2	7360	585
**8**	9800	110	12.1	>10,000	79.6	9120	2078
**9**	6500	40	34.9	>10,000	580	12,000	712
**10**	7300	54	54.3	>10,000	>50,000	23,500	350
**11**	5800	63	9	676	890	18,700	235
**12**	8400	75	9.2	>10,000	3350	>50,000	90
**13**	8600	60	7900	>10,000	8650	48.1	88
**14**	9300	19	8500	>10,000	7215	280	94
**15**	5500	80	23.6	>10,000	3160	143	605
**16**	9500	94	10.4	651	4520	92.5	82
**17**	21,000	125	6.3	>10,000	>50,000	390	507
**18**	164	46	6.8	>10,000	4443	3250	226
**19**	109	33	3500	779	475	6760	91
**20**	6	2	23.4	91	363	9880	85
**21**	69	11	11.8	740	4550	4060	95
**22**	164	46	9.4	374	1985	25,200	85
**23**	109	33	4530	>10,000	282	>50,000	89
**24**	95	30	256	>10,000	294	>50,000	84
**AAZ**	250	12	7600	10	445	816	94
**MZA**	50	14	7455	390	421	8140	91
**EZA**	25	8	3800	379	440	3170	95
**DCP**	1200	38	34.6	306	1220	5790	73
**DZA**	50,000	9	7900	81	360	742	274
**BRZ**	45,000	3	8400	70	451	739	61
**BZA**	15	9	48.2	715	2115	410	54
**TPM**	250	10	146	383	414	673	363
**ZNS**	56	35	765	>10,000	1820	1885	710
**SLP**	1200	40	32	485	1715	670	493
**IND**	31	15	n.d.	87	4240	216	94
**VLX**	54,000	43	3150	77	4425	3730	831
**CLX**	50,000	21	3480	140	2513	857	669
**SLT**	374	9	n.d.	67	3210	496	4838
**SAC**	18,540	5959	n.d.	620	5280	7075	191
**HCT**	328	290	n.d.	850	3350	6680	545
**FAM**	n.d.	n.d.	n.d.	>10,000	n.d.	n.d.	n.d.
**EPA**	n.d.	n.d.	n.d.	n.d.	n.d.	n.d.	n.d.

* Errors were in the range of ±5–10% on three different assays. ^1^ From reference [83] and [84]; ^2^ From reference [85]; n.d.: not detected.

**Table 2:** Inhibition constants obtained using anionic inhibitors versus the α-CA isozymes of human origin (hCA I and hCA II), and Table 1. CAS2, CAS3; for the acronyms, see the text) by a stopped flow CO_2_ hydrase assay.

Anion	K_I_ (mM) *						
	hCA I ^1^ (α-CA)	hCA II ^1^ (α-CA)	MgCA ^2^ (β-CA)	MreCA ^2^ (β-CA)	CAS1 ^1^ (β-CA)	CAS2 ^1^ (β-CA)	CAS3 ^1^ (β-CA)
F^−^	>300	>300	7.13	>50	>100	>100	>100
Cl^−^	6	200	7.98	>50	9.2	>100	>100
Br^−^	4	63	18.6	>50	9.3	>100	>100
I^−^	0.3	26	8.73	8.6	8.6	7.7	9.9
CNO^−^	0.0007	0.03	6.81	>50	0.9	0.82	3.2
SCN^−^	0.2	1.60	8.39	>50	5.4	5.6	7.3
CN^−^	0.0005	0.02	7.19	>50	0.94	0.75	8.7
N_3_^−^	0.0012	1.51	45.2	>50	>100	6.1	7.2
NO_2_^−^	8.4	63	7.56	>50	>100	>100	8.3
NO_3_^−^	7	35	8.13	9	>100	>100	8.5
HCO_3_^−^	12	85	0.59	0.86	3.3	7.3	>100
CO_3_^2−^	15	73	>100	>50	>100	8.8	8
HSO_3_^−^	18	89	>100	>50	3.3	7.3	>100
SO_4_^2−^	63	>200	19.5	>50	>100	4.8	>100
HS^−^	0.0006	0.04	11.9	>50	0.89	8.5	8.3
SnO_3_^2−^	0.57	0.83	5.07	0.56	4.3	0.92	7.9
SeO_4_^2−^	118	112	7.41	1.7	2.4	9.2	3.4
TeO_4_^2−^	0.66	0.92	5.75	0.56	2.5	6.3	8.1
OsO_5_^2−^	0.92	0.95	6.16	8.5	n.d.	n.d.	n.d.
P_2_O_7_^4−^	25.77	48.50	6.03	>50	3.1	0.96	>100
V_2_O_7_^4−^	0.54	0.57	6.89	>50	>100	1.4	>100
B_4_O_7_^2−^	0.64	0.95	8.45	0.4	6.7	6.9	5.9
ReO_4_^−^	0.11	0.75	16.7	>50	8.2	>100	8.8
RuO_4_^−^	0.101	0.69	8.82	7.4	3.9	>100	9.2
S_2_O_8_^2−^	0.107	0.084	>100	>50	5	>100	>100
SeCN^−^	0.085	0.086	1.73	0.65	2.9	9.3	7.1
CS_3_^2−^	0.0087	0.0088	1.77	0.92	0.79	>100	8.6
Et_2_NCS_2_^−^	0.00079	0.0031	0.30	0.075	0.38	0.93	0.89
CF_3_SO_3_^−^	n.d.	n.d.	2.28	4.5	n.d.	n.d.	n.d.
PF_6_^−^	n.d.	n.d.	6.47	3.9	n.d.	n.d.	n.d.
ClO_4_^−^	>200	>200	>100	9.2	>100	>100	>100
BF_4_^−^	>200	>200	>100	383	>100	>100	>100
FSO_3_^−^	0.79	0.46	4.06	>50	0.93	8.4	>100
NH(SO_3_)_2_^2−^	0.31	0.76	21.4	>50	0.88	9.2	>100
H_2_NSO_2_NH	0.31	1.13	0.094	0.72	0.084	0.048	0.094
H_2_NSO_3_H	0.021	0.39	0.083	7.7	0.069	0.072	0.095
Ph-B(OH)_2_	58.6	23.1	0.089	8.7	0.009	0.056	0.097
Ph-AsO_3_H_2_	31.7	49.2	0.090	0.83	0.035	0.054	0.091

* Errors were in the range of ±5–10% on three different assays. ^1^ From reference [87]; ^2^ From reference [88]; n.d.: not detected.

## Data Availability

Not applicable.
